# Mutations within the conserved NS1 nuclear export signal lead to inhibition of influenza A virus replication

**DOI:** 10.1186/1743-422X-11-128

**Published:** 2014-07-14

**Authors:** Janne Tynell, Krister Melén, Ilkka Julkunen

**Affiliations:** 1 Virology Unit, Department of Infectious Disease Surveillance and Control, National Institute for Health and Welfare (THL), Mannerheimintie 166, FIN-00300 Helsinki, Finland; 2 Department of Virology, University of Turku, Turku, Finland

**Keywords:** NS1, NES, Influenza A virus

## Abstract

**Background:**

The influenza A virus NS1 protein is a virulence factor and an antagonist of host cell innate immune responses. During virus infection NS1 protein has several functions both in the nucleus and in the cytoplasm and its intracellular localization is regulated by one or two nuclear localization signals (NLS) and a nuclear export signal (NES).

**Methods:**

In order to investigate the role of NS1 NES in intracellular localization, virus life cycle and host interferon responses, we generated recombinant A/Udorn/72 viruses harboring point mutations in the NES sequence.

**Results:**

NS1 NES was found to be inactivated by several of the mutations resulting in nuclear retention of NS1 at late stages of infection confirming that this sequence is a *bona fide* functional NES. Some of the mutant viruses showed reduced growth properties in cell culture, inability to antagonize host cell interferon production and increased p-IRF3 levels, but no clear correlation between these phenotypes and NS1 localization could be made. Impaired activation of Akt phosphorylation by the replication-deficient viruses indicates possible disruption of NS1-p85β interaction by mutations in the NES region.

**Conclusion:**

We conclude that mutations within the NS1 NES result in impairment of several NS1 functions which extends further from the NES site being only involved in regulating the nuclear-cytoplasmic trafficking of NS1.

## Background

Influenza A virus has a segmented genome consisting of eight single stranded negative-sense RNA molecules, which encode for 14 different proteins. Some of these proteins, such as the HA, PB1-F2 and NS1 proteins contribute to the virulence of the virus [1]. The 26 kDa NS1 protein is a remarkably multifunctional protein. Through protein-RNA and protein-protein interactions NS1 is capable of preventing the production of interferons in virus-infected host cells and inhibiting the antiviral actions of interferon-induced proteins (reviewed by [2]). The inhibition of interferon production is facilitated by the ability of NS1 to inhibit the functions of a cytoplasmic RNA sensor RIG-I through interaction with TRIM25 [3]. In addition, the C-terminal effector domain of NS1 can bind and inhibit the nuclear proteins CPSF30 and PABII, which function in the 3′-end processing of cellular pre-mRNAs [4,5]. The inhibition of interferon-induced dsRNA-activated proteins PKR and 2′-5′-oligoadenylate synthetase (OAS) is mediated through direct binding of NS1 to PKR [6] and through sequestering of dsRNA from OAS by the N-terminal RNA-binding domain of NS1 [7]. Moreover, NS1 has been suggested to inhibit the host RNAi pathway [8] and it is also involved in many regulatory functions such as controlling the host cell splicing machinery [9,10], enhancing the translation of viral mRNA [11] and activating the PI3K pathway [12].

Since NS1 has many functions both in the nucleus and in the cytoplasm, it is clear that the intracellular localization of NS1 must be controlled accordingly. Structural and functional studies of the NS1 protein have recognized an N-terminal nuclear localization signal (NLS1) involving amino acids Arg-35, Arg-38 and Lys-41 [13], a nuclear export signal (NES) within amino acids 138–147 [14] and a C-terminal nuclear/nucleolar localization signal (NLS2/NoLS) comprising of the basic amino acids at positions 219, 220, 224 and 229 and including also amino acids 231 and 232 in NS1 proteins which have a C-terminal extension [15,16]. Localization studies have shown that NS1 localizes predominantly in the nucleus, but it is translocated into the cytoplasm at late stages of infection possibly due to the unmasking of NES or, in the event of a competitive relationship between NLS and NES, perhaps because of masking of the NLS signals.

All NS1 proteins have a putative well-conserved NES, which is characterized as a classical hydrophobic leucine-rich export signal consisting of the consensus sequence Φ-XX(X)-Φ-XX(X)-Φ-X-Φ, where Φ represents L, I, F, V or M and X represents any amino acid. Leucine-rich NESs are typically recognised by the chromosome region maintenance 1 (CRM1) exportin molecule, which directly binds to NES and mediates the transport of NES-containing molecules through the nuclear pore complex in a NES-CRM1-Ran-GTP ternary complex (reviewed by [17]). Recently it was suggested that some leucine-rich NES-containing proteins may be translocated into the cytoplasm independently of CRM1 [18,19]. Accordingly, a FRET analysis of transiently expressed NS1 protein failed to demonstrate a direct interaction of NS1 with CRM1 [20]. However, since the NES of ectopically expressed NS1 has been reported to remain inactive in uninfected cells [14], this observation has to be verified in the context of live virus infection.

In the present study we generated recombinant influenza A viruses with mutations in the putative NES of NS1 protein. We show that mutations in the NES of NS1 molecule results in nuclear retention of the NS1 protein at late stages of infection. In addition, we obtained mutant viruses that had a reduced capacity to replicate in cell culture and showed enhanced activation of IRF3 and production of antiviral interferons (IFN). Lack of efficient Akt phosphorylation by the growth-deficient viruses suggests impairment of the NS1-p85β interaction by mutations within the NES.

## Results

### The NS1 nuclear export signal is well-conserved within the majority of influenza A virus strains

**Figure 1:**
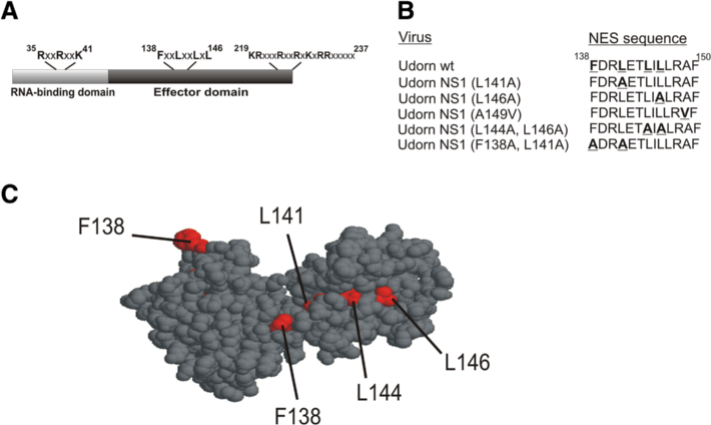
**The nuclear export signal of A/Udorn/72 NS1. A**, schematic representation of A/Udorn/72 NS1 with the localization signals NLS1 (aa 35–41), NES (aa 138–146) and NLS2/NoLS (aa 219–232) indicated. **B**, sequence of A/Udorn/72 NS1 NES region and the mutations introduced into the NES sequence to create recombinant mutant viruses. **C**, molecular surface representation of the position of NES within the dimericA/Udorn/72 NS1 effector domain based on the crystal structure by Xia et al. [22] (PDB ID 3EE8). Amino acids of the NES consensus sequence are indicated in red. The figure was created using RasMol 2.7.3. program (http://rasmol.org/).

A schematic presentation of the NS1 protein structure (A/Udorn/72 virus NS1) is shown in Figure 1. The NS1 NES is located in the middle of the effector domain and comprises of a phenylalanine and three leucines each separated by one or two amino acids (Figure 1A). Within the dimeric structure of the effector domain the NES sequences are partially buried in the molecule (Figure 1C). In order to determine the conservation rate of the NS1 NES we compared more than 25,000 influenza A NS1 amino acid sequences available (as of September 2013) in the Influenza Research Database [21] using the sequence variation analysis tool accessible on the website http://www.fludb.org (Table 1). The NES consensus sequence (F138, L141, L144 and L146) appears to be highly conserved within all influenza A strains regardless of host species. In addition, the region immediately adjacent to the export signal (aa 147–150) is also very highly conserved.

**Table 1:** Amino acid conservation of the NS1 NES region

**Position**	**Consensus**	**Prevalence**	**Percentage**
138	Phe	24424/25183	96,99%
139	Asp	15412/25183	61,20%
140	Arg	22525/25183	89,44%
141	Leu	24763/25183	98,33%
142	Glu	24916/25183	98,94%
143	Thr	22149/25183	87,95%
144	Leu	21822/25183	86,65%
145	Ile	16910/25183	67,15%
146	Leu	22655/25183	89,96%
147	Leu	25105/25183	99,69%
148	Arg	25099/25183	99,67%
149	Ala	25134/25183	99,81%
150	Phe	25092/25183	99,64%

A consensus sequence for the region was derived by running 25183 NS1 sequences through the Analyze Sequence Variation (SNP) tool on the Influenza Research Database –website (http://www.fludb.org). Prevalence of each amino acid is indicated as a total number and percentage within all the analyzed sequences.

### Mutations in the putative nuclear export signal lead to nuclear retention of NS1 at late stages of infection

**Figure 2:**
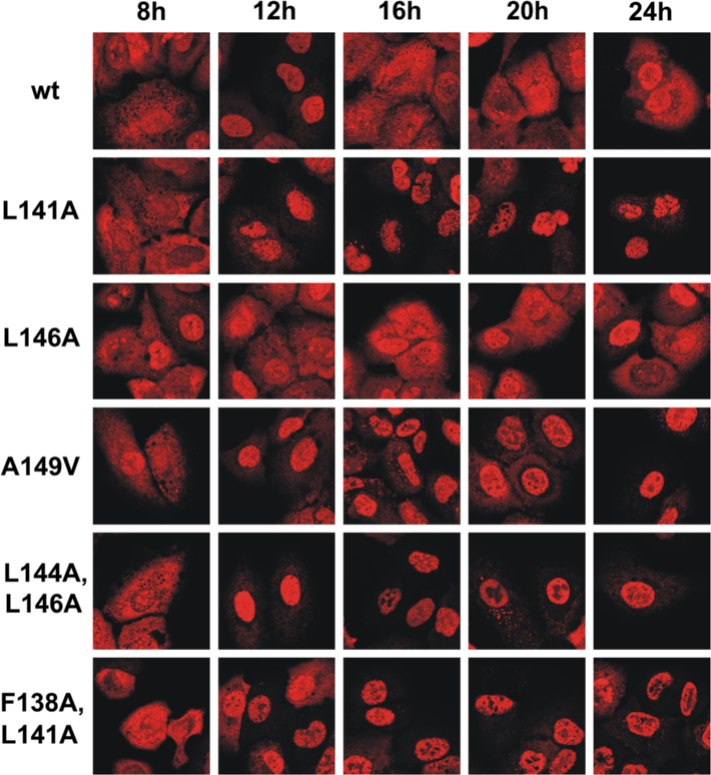
**Nuclear-cytoplasmic localization of wild-type and mutant NS1 proteins in A549 cells during infection.** A549 cells were infected with the recombinant A/Udorn/72 viruses at MOI 1 for times indicated in the figure followed by fixation with 3% paraformaldehyde. Cells were permeabilized, stained with guinea pig anti-NS1 and secondary antibodies and processed for detection by immunofluorescence microscopy. The pictures were taken with Leica TCS NT confocal microscope. Data shown is representative of three independent experiments.

**Figure 3:**
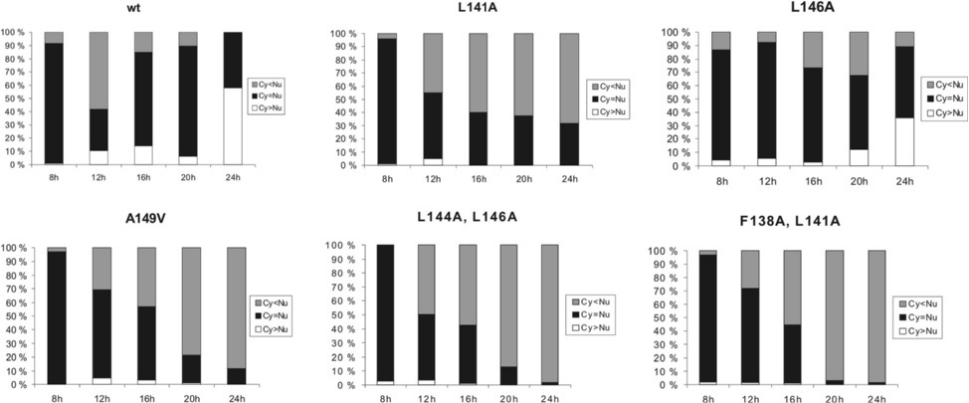
**Localization patterns of wild-type and mutant NS1 proteins in A549 cells.** A549 cells were infected with different recombinant A/Udorn/72 viruses for times indicated after which they were fixed, permeabilized and processed for detection by immunofluorescence. For each time point five random frames under the fluorescence microscope (≈100 cells) were inspected and the intracellular localization of NS1 protein was estimated and presented in a quantitative manner. Data shown is from the same samples as the immunofluorescence pictures in Figure 2.

In order to determine the key amino acids regulating the nuclear export of NS1 protein we created different A/Udorn/72 mutant viruses with one or two point mutations introduced into the putative NES sequence (Figure 1B). In addition, we also generated NS1 A149V mutant virus, since this mutation has been linked to an attenuated phenotype in an avian influenza A virus [23]. The impact of the putative NES mutations on NS1 intracellular localization at different stages of infection was studied in A549 cells by immunofluorescence assay (Figure 2) followed by quantitative analysis of the intracellular localization of wild type and mutant NS1 proteins (Figure 3). At early stages of infection there were no marked differences between the localization patterns of the mutated proteins and the wild-type NS1 protein, since in all cases the protein was found both in the nucleus and the cytoplasm. At later stages of infection (16 h and later), however, most of the mutant NS1 proteins (L141A), (A149V), (F138A, L141A) and (L144A, L146A) accumulated in the nucleus leaving the cytoplasm almost or completely devoid of NS1 protein, while a significant proportion of the wild-type NS1 protein localized into the cytoplasm in addition to the nucleus. The NS1 L146A mutant protein behaved very similarly to that of the wt NS1 protein and thus this point mutation appears to be insufficient to fully inactivate the NES (Figures 2 and 3).

### Inactivation of the NS1 NES has limited impact on replication of the virus

**Figure 4:**
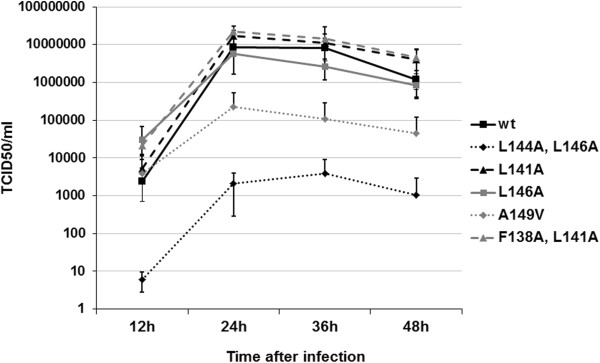
**Growth kinetics of the recombinant viruses in MDCK cells.** MDCK cells on 6-well plates were infected at MOI values of 0.001 for 1 h after which virus inoculum was removed and fresh growth medium was added. The cells were incubated at 37°C humidified 5% CO_2_ conditions and supernatant samples were taken at 12 h intervals and analyzed by endpoint dilution on MDCK cells. Mean values from three independent experiments are shown for each sample.

**Figure 5:**
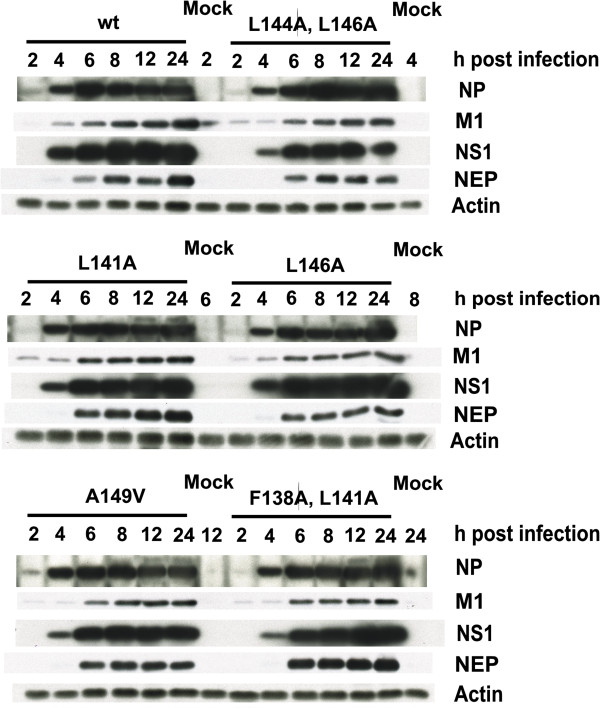
**Expression kinetics of viral proteins during infection.** A549 cells were infected at MOI 1 with A/Udorn/72 wild type and mutant viruses and cell lysates were collected at the indicated time points. The amounts of NS1, NP, NEP, M1 and actin in the lysates were visualized by immunoblotting (10 μg total protein/lane). Data shown is representative of three independent experiments.

Next we analyzed the effect of NES mutations on the viral growth properties of different recombinant viruses. We infected MDCK cells with the wt and NES-mutant viruses at very low MOI values (0.001) and measured the virus titers from samples taken at 12 h intervals. The growth curves derived from the results showed no major differences in mutant virus titers as compared to the wild type virus, with the exception of the (A149V) and (L144A, L146A) NS1 mutant viruses (Figure 4). The growth of (L144A, L146A) NS1 mutant virus was strongly inhibited, presenting approximately 1000-fold lower titers throughout the infection than the wt virus. Also the growth kinetics of (L144A, L146A) NS1 mutant virus was delayed with peak titer achieved only at 36 h after infection as compared to 24 h with all the other viruses. The (A149V) NS1 mutant virus displayed equal titers with wt virus at the 12 h time point, but afterwards only grew to 10–100 fold lower titers than the wt virus. The observed reduction in virus production is not directly linked with the inactivation of the NES, since (L141A) and (F138A, L141A) NS1 mutant viruses show clear inactivation of NES and accumulation of NS1 protein in the nucleus (Figure 2) and yet they replicated very well in MDCK cells. To investigate whether the observed growth defects correlated with any deficiencies in viral protein expression, we infected A549 cells at MOI 1 and analyzed the expression of NP, NS1, M1 and NEP in cell lysate samples collected at 2 h, 4 h, 6 h, 8 h, 12 h and 24 h post-infection by Western blotting (Figure 5). All viruses displayed largely similar viral protein expression kinetics throughout the infection (Figure 5), but there was a clear reduction in the levels of NS1 and NEP production by the (L144A, L146A) mutant virus and some less pronounced variation among the other viruses. NEP mediates viral ribonucleoprotein (vRNP) export [24], so we checked for any differences in vRNP localization by visualizing NP localization by immunofluorescence in infected A549 cells (Additional file 1: Figure S1 and Additional file 2: Figure S2), but found no differences between the viruses.

### The NS1 mutations A149V and L144A, L146A lead to increased interferon production

**Figure 6:**
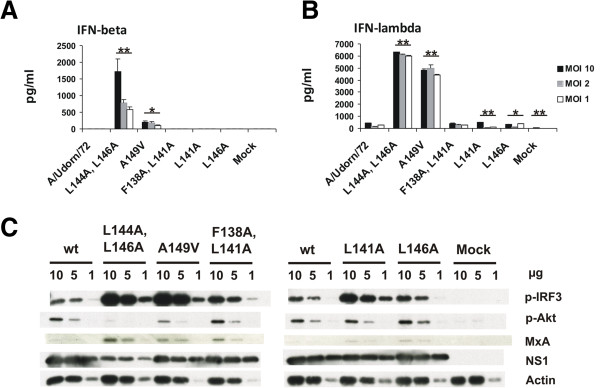
**The effect of NS1 NES mutations on recombinant virus-induced interferon production and IRF3 and Akt phosphorylation.** A549 cells on 6-well plates were infected at three different MOI values for 20 h after which the supernatants and cell lysates were collected. **A** and **B**, quantitation of IFN-β and IFN-λ1 from the supernatants by ELISA. All samples were analyzed in duplicates and the data shown is representative of three independent experiments. Statistical significance is calculated against the wt samples (*p < 0.05, **p < 0.01). **C**, Virus-infected cells (MOI 1) were collected at 20 h postinfection, lysed and processed for SDS-PAGE and Western blotting in three different dilutions (10, 5 or 1 μg of total protein/well). Filters were stained with antibodies against p-IRF3, p-Akt, MxA, actin and NS1 proteins and relative protein expression is shown in the figure. Data shown is representative of three independent experiments.

In order to analyse whether the reduced replication of (A149V) and (L144A, L146A) mutant viruses is the result of an impaired ability of NS1 protein to block host cell interferon production, we infected A549 cells with the 5 mutant viruses and the wild-type virus at different MOI values and measured the production of IFN-β and IFN-λ1 into the supernatants at 20 h post infection (Figure 6A and B). Recombinant wild type A/Udorn/72 virus and NS1 (L141A), (L146A) and (F138A, L141A) mutant viruses were very weak in their ability to induce IFN-β and IFN-λ1 production into the cell culture supernatant. However, NS1 (A149V) and especially NS1 (L144A, L146A) mutant viruses readily induced relatively high IFN levels in response to the infection (Figure 6A and B). Consistent with the ELISA data, Western blot analysis of cell lysates from virus-infected cells showed high levels of phosphorylated form of IRF3 (p-IRF3) and enhanced expression of MxA protein for the NS1 (A149V) and (L144A, L146A) viruses (Figure 6C). Interestingly, NS1 (L141A), (L146A) and (F138A, L141A) mutant viruses were also able to induce stronger IRF3 phosphorylation than the wt virus and detectable levels of MxA (Figure 6C), even though only low levels of IFN were produced by these viruses (Figure 6A and B). Since the phosphaditylinositol-3-kinase (PI3K) pathway is also activated and regulated by NS1 protein during influenza virus infection [12], we analyzed whether NES mutations would also have an effect on Akt phosphorylation. Interestingly, the amount of p-Akt within the infected cells was clearly reduced in the NS1 (A149V) and (L144A, L146A) mutant virus infected cells, suggesting an impairment of the PI3K activating functions of NS1. A notable feature was also the clear reduction in NS1 levels for the mutant virus (L144A, L146A) (Figure 6C), indicating a possible impairment of protein stability caused by the mutations. For better comparison of NS1 expression levels, a new Western blot analysis with further diluted samples was performed and band intensities were analyzed with ImageJ (http://imagej.nih.gov/ij/) software, revealing an almost 50% reduction in NS1 (L144A, L146A) expression levels as compared to wild type virus (Additional file 3: Figure S3).

### Interferon production by A149V and L144A, L146A mutant viruses is not due to inability of NS1 to bind CPSF

**Figure 7:**
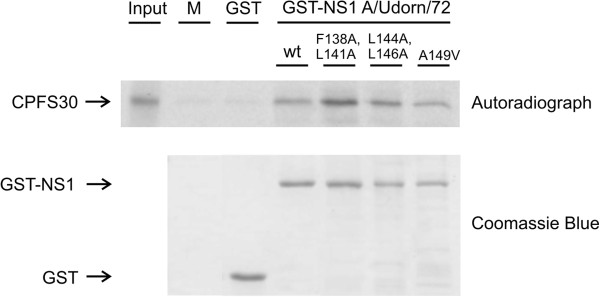
**The nuclear export -deficient mutants of the full length influenza A/Udorn/72 NS1 protein bind *in vitro*-translated CPFS30 protein in a GST pull down experiment.** [^35^S]-labeled and *in vitro*-translated CPFS30 was allowed to bind to *E. coli*-expressed and Sepharose-immobilized GST, GST-NS1 A/Udorn/72 wt, GST-NS1 F138A, L141A, GST-NS1 L144A, L146A and GST-NS1 A149V at +4°C for 1 h. Sepharose-bound proteins were dissolved in Laemmli sample buffer, separated on 12% SDS-PAGE and autoradiographed. Input, lane C, was 1:20 of the amount of [^35^S]-labeled protein that was used in binding experiment. M indicates the Sepharose matrix-bound proteins. The same samples were also separated on 12% SDS-PAGE and stained with Coomassie Blue to visualize the amount of Sepharose-immobilized GST and GST-NS1 fusion proteins.

The NES-region of NS1 has also been proposed to play a role in CPSF binding [25]. In order to study whether the observed increase in interferon production was due to an inability of the NS1 protein to bind CPSF, we created the mutations (A149V), (L144A, L146A) and (F138A, L141A) into GST-NS1 expression constructs and compared their ability to bind CPSF30 to that of wild-type NS1 in a GST pull-down assay combined with autoradiography (Figure 7). As shown in Figure 7, all the mutant GST-NS1 proteins bind CPSF30 equally well and thus we can conclude that the NES mutations are unlikely to have an effect on NS1 CPSF-binding.

### NS1 protein export during influenza A infection is likely not mediated by CRM1

**Figure 8:**
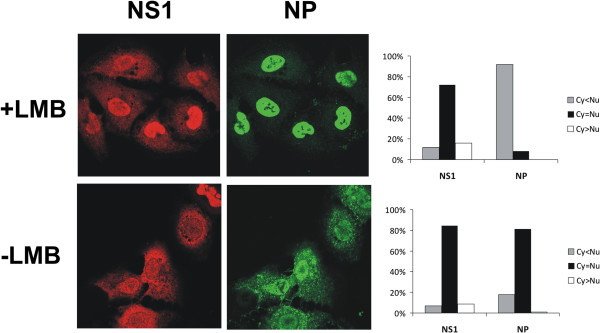
**Effect of leptomycin B treatment on NS1 intracellular localization.** A549 cells were infected with wild-type A/Udorn/72 virus and incubated with or without 2 ng/ml LMB added at 3 h postinfection. 20 h postinfection the cells were fixed and the intracellular localization of NS1 protein and NP were visualized by immunofluorescence and estimated in a quantitative manner by observing 6–8 random frames under the microscope. The pictures were taken by Leica TCS NT confocal microscope. Data shown is representative of three independent experiments.

The nuclear export of NES-containing proteins is often regulated by the nuclear export protein, CRM1. An antifungal antibiotic, leptomycin B (LMB) is able to directly bind to CRM1 [26] and thus block NES-dependent nuclear export of proteins. In order to analyze whether the nuclear export of influenza A virus NS1 protein is dependent on CRM1 we treated wt virus-infected cells with LMB and analyzed the intracellular localization of NS1 protein by immunofluorescence at 20 h post infection (Figure 8). As a positive control we included the analysis of viral NP, since its export from the nucleus has previously been shown to be dependent on CRM1 [27]. For a quantitative assessment 6–8 random frames (≈100 cells) were inspected under the microscope and NS1 and NP localization was estimated and presented in a graph (Figure 8). NP was clearly found to be retained in the nucleus in LMB-treated cells confirming the successful inhibition of CRM1 functions. NS1, however, showed little difference in localization between LMB- and mock-treated cells with most cells displaying NS1 both in the nucleus and the cytoplasm. This suggests that LMB treatment was inefficient in inhibiting the nuclear export of NS1 indicating that the export of this protein is likely independent or only partially dependent on CRM1.

## Discussion

In the present study we have carried out a detailed functional analysis of influenza A virus NS1 protein NES element, which is extremely well-conserved among all presently known influenza A virus strains and represents a typical consensus NES-type sequence. We show that mutations in the NES element, with the exception of mutation L146A, lead to nuclear retention of NS1 at late stages of infection, indicating an inactivation of the NES. NES-inactivating NS1 mutations (L144A, L146A) and (A149V) were associated with severe inhibition of viral growth properties and inability of the mutant virus to inhibit host cell interferon production.

Regulation of the mechanisms of NS1 NES function during infection is unknown, although the amino acid region immediately after the consensus-like NES element has been suggested to play a regulatory role in the nuclear export of NS1 [14]. This may take place by masking of the export signal at early times of infection and by allowing the exposure of the NES element at later time points by some activating event. The fact that some activation event is required for triggering the nuclear export of NS1 protein is well supported by the structure of NS1 (Figure 1C). Leucine residues in the NES element are of hydrophobic nature and for them to become in contact with export molecules relatively drastic conformation changes are required. Inactivation of the NES by the A149V mutation, which renders the protein strongly nuclear at late time points (Figure 2), also supports the view that some masking sequence is regulating the accessibility of the consensus leucine residues to the export machinery. Interestingly, the A149V NS1 mutation has been linked to pathogenesis in chickens when it was discovered in the context of a natural H5N1 virus strain that was circulating in poultry [23].

The observed reduction in viral replication of the NS1 mutant viruses (A149V) and (L144A, L146A) (Figure 4) was likely due to impaired IFN antagonist functions of mutant NS1 proteins. These viruses readily induced IFN production into cell culture supernatant, as evidenced by the ELISA assays (Figure 6A and B), and also showed induction of IFN induced MxA protein (Figure 6C). Upon further investigation the elevated IFN levels were found to correlate with very high virus activated p-IRF3 levels as compared to wild-type virus, suggesting the inability of these mutant NS1 proteins to inhibit the RIG-I pathway. Strong expression of p-IRF3 was also observed with the other nuclearly localized NS1 mutant viruses (L141A) and (F138A, L141A), which might be explained by the nuclear NS1 being unable to inhibit cytoplasmic RIG-I. However, this correlation remains unclear since strong phosphorylation of IRF-3 was also associated with the L146A NS1 mutant virus, which shows a similar NS1 localization pattern as the wild type virus. Lack of potent IFN production despite strong p-IRF3 activation by the (L141A) and (F138A, L141A) NS1 mutant viruses indicates, that in the case of at least some nuclearly retented mutant NS1 proteins, the nuclear functions of NS1 in the absence of cytoplasmic NS1 are sufficient to inhibit IFN production. Detectable levels of MxA were observed with all the mutant viruses despite low IFN production by some of them, which is probably due to even low IFN production being enough to cause some MxA production. It was also of interest that Western blot analysis revealed diminished p-Akt levels for the (L144A, L146A) and (A149V) mutants adding further evidence that multiple NS1 functions may be affected by the mutations. This suggests a more extensive structural or functional importance of the NES region. In a study investigating the structural conservation of the whole NS1 protein, Leu144 and Ala149 were found to be very well-conserved and were suggested to play a role in stabilizing the protein structure [28]. The observed reduction of almost 50% in the levels of NS1 (L144A, L146A) compared to wild type NS1 (Additional file 3: Figure S3) supports the idea of a stabilizing role for the NES region, but this is unlikely to have an impact on viral growth properties or IFN production since very low levels of NS1 have been shown to be sufficient for normal replication and IFN antagonism [29]. The NES region has also been suggested to be involved in CPSF binding [25], but an X-ray crystal structure published on the NS1-CPSF complex somewhat later does not support this view [30]. Nevertheless, we analyzed whether any of our inserted mutations affected CPSF-binding of NS1 on GST pull-down assay and found that binding to CPSF30 was similar to that of wild-type NS1 (Figure 7).

Besides being involved in regulating splicing and nuclear export of mRNA [10], NS1 has also been shown to influence temporal regulation of viral RNA and protein synthesis [31]. Thus we explored whether the introduced NES mutations might have an effect on viral protein expression kinetics (Figure 5). In addition to NS1, we included an abundant early gene product (NP), a late gene product (M1) and a spliced gene product (NEP). At a glance, expression kinetics of all the analyzed proteins appear similar between the wild type and mutant viruses (Figure 5), but there are some differences in expression levels at certain time points. The growth deficient viruses (A149V) and especially (L144A, L146A) produce somewhat lower levels of NS1 and NEP, whereas the (L141A) and (L146A) viruses are even stronger inducers of NEP than the wild type virus (Figure 5). NP and M1 expression levels are mostly equal between the different viruses with the exception of (A149V), (L146A) and (F138A, L141A) viruses showing slightly reduced M1 levels. All the observed differences are modest however, and despite critical roles for these proteins during infection, unlikely to cause any defects in replication. To confirm this conclusion we used immunofluorescence analysis to visualize NP localization during infection with all our mutant viruses (Additional file 1: Figure S1 and Additional file 2: Figure S2). Since NEP and M1 are involved in the nuclear export of viral ribonucleoproteins [24,32], any major flaw in their function would show as nuclear retention of NP. All the viruses display equal cytoplasmic localization of NP (Additional file 1: Figure S1 and Additional file 2: Figure S2), so we can conclude that the observed growth deficiencies are not due to any defects in viral mRNA splicing or protein expression.

The PI3K pathway has a dual role in influenza A virus infection by mediating processes that are both beneficial and detrimental for virus replication. Several studies have shown that the PI3K pathway supports viral replication by promoting viral entry [33], inhibiting apoptosis [34-36] and enhancing nuclear export of vRNP complexes [37]. In addition, PI3K signalling is also involved in RIG-I mediated activation of IRF3 thus contributing to the induction of type I interferon production [38]. PI3K is activated at two points during influenza A infection, first at a very early stage stimulated by viral attachment and then at a later stage of infection by NS1 binding to the p85β subunit of PI3K [33]. NS1 has also been suggested to directly bind to Akt, a downstream effector of PI3K involved in diverse cellular processes including cell differentiation, proliferation and apoptosis [39]. Diminished levels of cellular p-Akt observed in (L144A, L146A) and (A149V) mutant virus-infected cells point towards defects in the NS1 PI3K activating functions (Figure 6C), which correlates with a previous observation of the region around NES being involved in PI3K activation [40]. Li and coworkers found the amino acid region 137–142 to be involved in NS1-p85β interaction and showed interference of the interaction by mutation of the amino acids 141 and 142. In a structural study of the p85β iSH2-NS1 -complex the Glu 142 of NS1 was speculated to be involved in salt bridge formation at the complex interface [41]. The inability of NS1 (L144A, L146A) and (A149V) mutant viruses to activate PI3K signalling could result from NS1 nuclear retention, but since this phenotype is not shared by other viruses with inactivated NS1 NES signals, it must be due to an effect independent of NS1 localization. It is plausible that mutations close to the NS1-p85β binding site could cause structural changes big enough to disrupt the salt bridge formation between NS1 Glu 142 and p85β or just influence the interaction enough to disrupt PI3K/Akt activation. Such disruption by itself could cause the observed reductions in growth and IFN antagonistic properties as evidenced by other studies where NS1-p85β interaction has been investigated [12,41,42]. Also certain mutations in the amino acid position 138 have been shown to influence the NS1-p85β interaction [43,44], so it is feasible that the mutations L144A, L146A and A149V might affect it as well.

Nuclear export of proteins containing the classical leucine-rich export signal is usually mediated by CRM1, but with influenza A virus NS1 this is likely not the case. It was shown previously by transfection experiments that NS1 does not interact with CRM1 [20] and here we show that the CRM1 inhibitor LMB fails to prevent or at least partially impair NS1 export during the infection (Figure 8). Examples of proteins showing nuclear export independent of CRM1, despite harboring leucine-rich export signals, include protein kinase inhibitor (PKI) and glucocorticoid receptor, which are exported by the Ca^2+^-binding protein calreticulin through a mechanism similar to CRM1-mediated export [18,19]. It is likely that a similar alternative export pathway exists for NS1 as well, but this remains to be verified by further experiments.

## Conclusions

In conclusion, our data shows that the NES region of NS1 plays an extremely important role during influenza A virus infection. It was clearly shown that the NES sequence regulates the nucleo-cytoplasmic transport of NS1. An association with nuclear retention of NS1 and increased phosphorylation of IRF3 was observed, but no clear correlation could be established. There were obvious differences in the growth properties of the different NES mutant viruses, and since the observed NS1 localization pattern was a poor prognosis for the growth phenotype, it must be concluded that the importance of NS1 NES region goes beyond just regulating NS1 localization. Good correlation of Akt phosphorylation with growth properties indicates the involvement of NS1-p85β interaction, but conclusive identification of the molecular basis of these differences requires further studies.

## Methods

### Cells and viruses

HEK293, A549 and MDCK cells were maintained in Eagle’s minimum essential medium (MEM) supplemented with 0.6 μg/ml penicillin, 60 μg/ml streptomycin, 2 mM L-glutamine, 20 mM HEPES and 10% fetal calf serum (FCS) at 37°C in 5% CO_2_. Influenza A/Udorn/72 recombinant wild-type virus (wt) and recombinant NS1 NES -mutant viruses were propagated in 11-day-old embryonated chicken eggs at 34°C for 3 days.

### Generation of recombinant viruses

The wild-type A/Udorn/72 virus and the recombinant NS1 NES-mutant viruses were generated using plasmid-based reverse genetics essentially as previously described [45]. pHH21 plasmids containing the full-length cDNAs for the eight A/Udorn/72 genomic RNA segments and pcDNA plasmids coding for the A/Udorn/72 proteins PB1, PB2, PA and NP were kindly provided by Dr. Robert A. Lamb. NES-mutations (L141A), (L146A), (A149V), (F138A, L141A) and (L144A, L146A) were introduced into the pHH21-NS plasmid using specific oligonucleotide primers and a QuikChange II Site-Directed Mutagenesis Kit (Stratagene, La Jolla, CA). ORF NS2 was not affected by any of the mutations. For the generation of recombinant viruses a co-culture of HEK293 and MDCK cells was co-transfected with 1 μg of each of the 12 plasmids coding for the viral RNA segments and expression contructs using TransIT-LT1 transfection reagent (Mirus, Madison, WI). The transfected cells were maintained in serum-free MEM and at 24 h post-transfection 2,5 μg/ml TPCK-trypsin was added into the culture medium. At 3 days post-transfection culture supernatants were harvested and viruses were propagated through two passages followed by plaque purification on MDCK cells. Individual plaques were then amplified in 11-day-old embryonated chicken eggs. NS genes of the recombinant viruses were sequenced to verify that the generated mutations were as expected.

### Antibodies

Guinea pig anti-NS1 [15] and rabbit anti-NP antibodies [46] have been previously described. Rhodamine Red X-labeled anti-guinea pig immunoglobulins and FITC-labeled anti-rabbit immunoglobulins were used as secondary antibodies in immunofluorescence analysis (1:100, Jackson ImmunoResearch Laboratories, Inc., West Grove, PA, USA). In Western blotting guinea pig anti-NS1 [15], rabbit anti-Actin (sc-10731; 1:500 dilution; Santa Cruz Biotechnology, Santa Cruz, CA), rabbit anti-MxA [47], rabbit anti-p-IRF3 (#4947; 1:1000 dilution: Cell Signaling Technology, Inc., Beverly, MA, USA) and rabbit anti-p-Akt (#9271 s; 1:1000 dilution: Cell Signaling Technology,) immunoglobulins were used. For anti-M1 and anti-NEP antibodies, Glutathione S-transferase (GST) –tagged A/Udorn/72 GST-M1 and polyhistidine-tagged A/Udorn/72 His-NEP (aa 11–121) were expressed in *Escherichia coli*, purified with Glutathione-Sepharose (Amersham Biosciences, Buckinghamshire, United Kingdom) or preparative SDS-PAGE, respectively, and used to immunize rabbits four times at 3-week intervals (100 μg or 60 μg of antigen/immunization, respectively). As secondary antibodies in Western blot HRP-conjugated rabbit anti-guinea pig (1:1000 dilution; Dako, Glostrup, Denmark) and goat anti-rabbit (1:2000 dilution; Dako) immunoglobulins were used.

### Immunofluorescence analysis

A549 cells grown on glass coverslips were infected with the wt or NES-mutant viruses at a multiplicity of infection (MOI) -value of 1 and incubated at 37°C 5% CO_2_ for 1 h, after which the virus inoculum was removed and replaced with MEM containing 2% FCS. Incubation was resumed and at 8 h, 12 h, 16 h, 20 h and 24 h post-infection infected cells were fixed with 3% paraformaldehyde at room temperature for 20 min, permeabilized with 0,1% Triton X-100 for 5 min and processed for immunofluorescence microscopy as described previously [48]. The localization of NS1 and NP in the cells were visualized with anti-NS1 and anti-NP antibodies (1:100 dilution) and photographed on a Leica TCS NT confocal microscope. For the leptomycin B (LMB) experiment 2 ng/ml of LMB was added on A549 cells infected with wt virus at 3 h post-infection and the cells were fixed at 20 h post-infection and processed as described above.

### Viral growth kinetics

Confluent monolayers of MDCK cells were inoculated with the wt or NES-mutant viruses at MOI values of 0.001 and incubated at 37°C 5% CO_2_. At 1 h post-infection the virus suspensions were removed and replaced with serum-free MEM containing 2,5 μg/ml TPCK-trypsin. Incubation was resumed and cell culture supernatant samples were collected at 12 h intervals until 48 h post-infection. Virus titers of the samples were measured by endpoint dilution assay on MDCK cells as per standard protocol. Briefly, serial ten-fold dilutions till 10^−8^ of each sample was made and used to infect confluent MDCK-cells on 96-well plates maintained in serum-free MEM with 2,5 μg/ml TPCK-trypsin. Eight parallel wells for each dilution were used. After three days of incubation at 37°C 5% CO_2_ the wells were inspected for cytopathic effect by light microscope and TCID_50_/ml –values were calculated using the Spearman-Karber method.

### Infection experiments

For analyzing interferon (IFN) gene expression A549 cells on 6-well plates were infected at MOI values of 10, 2 and 1 for 1 h followed by removal of the virus inoculum and addition of fresh medium (2 ml/well). At 20 h post-infection cell culture supernatants were collected and IFN-β and IFN-λ1 levels were determined by IFN-specific EIA assays using commercial kits (PBL Interferon source) according to the instructions of the manufacturer. Infected cells were collected in 1 mM EDTA, 10 mM Tris–HCl and lysed by pulling through a 25G needle ten times. Protein concentrations of the MOI 1 lysates were analysed by the Bradford method and equal amounts of proteins at different dilutions (10, 5, 1, 0,5 or 0,25 μg of total protein/lane) were loaded and separated on 12% SDS-PAGE. Proteins were transferred onto Immobilon P polyvinylidene fluoride membranes, stained (1 h at RT or overnight +4°C for p-IRF3 and p-Akt) with antibodies against NS1, Actin, MxA, p-IRF3 and p-Akt followed by staining with HRP-conjugated secondary antibodies for 1 h at RT. Protein bands were visualized on HyperMax films using an ECL plus system (GE Healthcare). Statistical significance of the IFN-production results were calculated by comparing samples to the corresponding wild-type virus samples using the students t-test. For analysis of viral protein expression kinetics, A549 cells were infected at MOI 1 as described above and cell lysates were collected at 2 h, 4 h, 6 h, 8 h, 12 h and 24 h post-infection and immunoblotted against NS1, NP, M1, NEP and Actin as described above.

### NS1 binding assay

The full length wild type A/Udorn/72 NS1 gene (V01102) was expressed in *E. coli* GST (pGEX-3X; Amersham Biosciences, Buckinghamshire, U. K.) expression vector. To create point mutations to NS1 cDNA, QuikChange™ Site-Directed Mutagenesis Kit (Stratagene, La Jolla, CA, USA) was used. All DNA manipulations were performed according to standard protocols or as specified by the manufacturer, and the newly created gene constructs were sequenced. Influenza A virus GST-NS1 fusion proteins were expressed in *E. coli* BL21 cells, and GST-fusion proteins were purified in Glutathione-Sepharose as described [15]. *In vitro*-translated CPSF30 protein (TnT Coupled Reticulocyte Lysate Systems, Promega, Madison, WI, USA) were [^35^S]-labeled (PRO-MIX, Amersham Biosciences) and allowed to bind to Sepharose-immobilized GST or GST-NS1 fusion proteins on ice for 1 h, followed by washing. GST-NS1-bound and [^35^S]-labeled proteins were separated on 12% SDS-PAGE. The gels were fixed and treated with Amplify reagent (Amersham Biosciences, Buckinghamshire, U. K.) as specified by the manufacturer and autoradiographed.

## Competing interests

The authors declare that they have no competing interests.

## Authors’ contributions

JT did the experiments, participated in the design and wrote the manuscript. KM gave hands-on guidance on the experiments and participated in the design of the experiments and writing of the manuscript. IJ participated in the design of the experiments and writing of the manuscript. All authors read and approved the final manuscript.

## Supplementary Material

Additional file 1: Figure S1 Intracellular localization of NP and NS1 on A549 cells. A549 cells were infected at MOI 1 for the times indicated with wild type, (L141A) and (L146A) recombinant viruses before fixation and permeabilization. Cells were stained with guinea pig anti-NS1 and rabbit anti-NP antibodies followed by Rhodamine Red X-labeled anti-guinea pig immunoglobulins and FITC-labeled anti-rabbit immunoglobulins. Pictures were taken with Leica TCS NT confocal microscope. Click here for file

Additional file 2: Figure S2 Intracellular localization of NP and NS1 on A549 cells. A549 cells were infected at MOI 1 for the times indicated with (A149V), (L144A, L146A) and (F138A, L141A) recombinant viruses before fixation and permeabilization. Cells were stained with guinea pig anti-NS1 and rabbit anti-NP antibodies followed by Rhodamine Red X-labeled anti-guinea pig immunoglobulins and FITC-labeled anti-rabbit immunoglobulins. Pictures were taken with Leica TCS NT confocal microscope. Click here for file

Additional file 3: Figure S3 Comparison of NS1 expression levels. A, lysates collected from infected A549 cells (MOI 1) at 20 h post-infection were analyzed by SDS-PAGE and Western blotting and the amounts of NS1 and actin were visualized by immunoblotting. Shown are three dilutions for each sample (10, 1, 0,5 or 0,25 μg total protein/lane). B, bands from the 1 μg lanes were processed with ImageJ software (http://imagej.nih.gov/ij/) to turn pixel intensity into optical density (OD). NS1 OD values were normalized to actin OD values and compared to wild type virus to get a relative estimate of NS1 expression levels between the viruses. Data shown is representative of two independent experiments. Click here for file
