# Supplementary figures and images for: Mutations within the conserved NS1 nuclear export signal lead to inhibition of influenza A virus replication
Source: Virol J. 2014 Jul 14;11:128. doi: 10.1186/1743-422X-11-128 (PMC4112715; doi:10.1186/1743-422X-11-128)

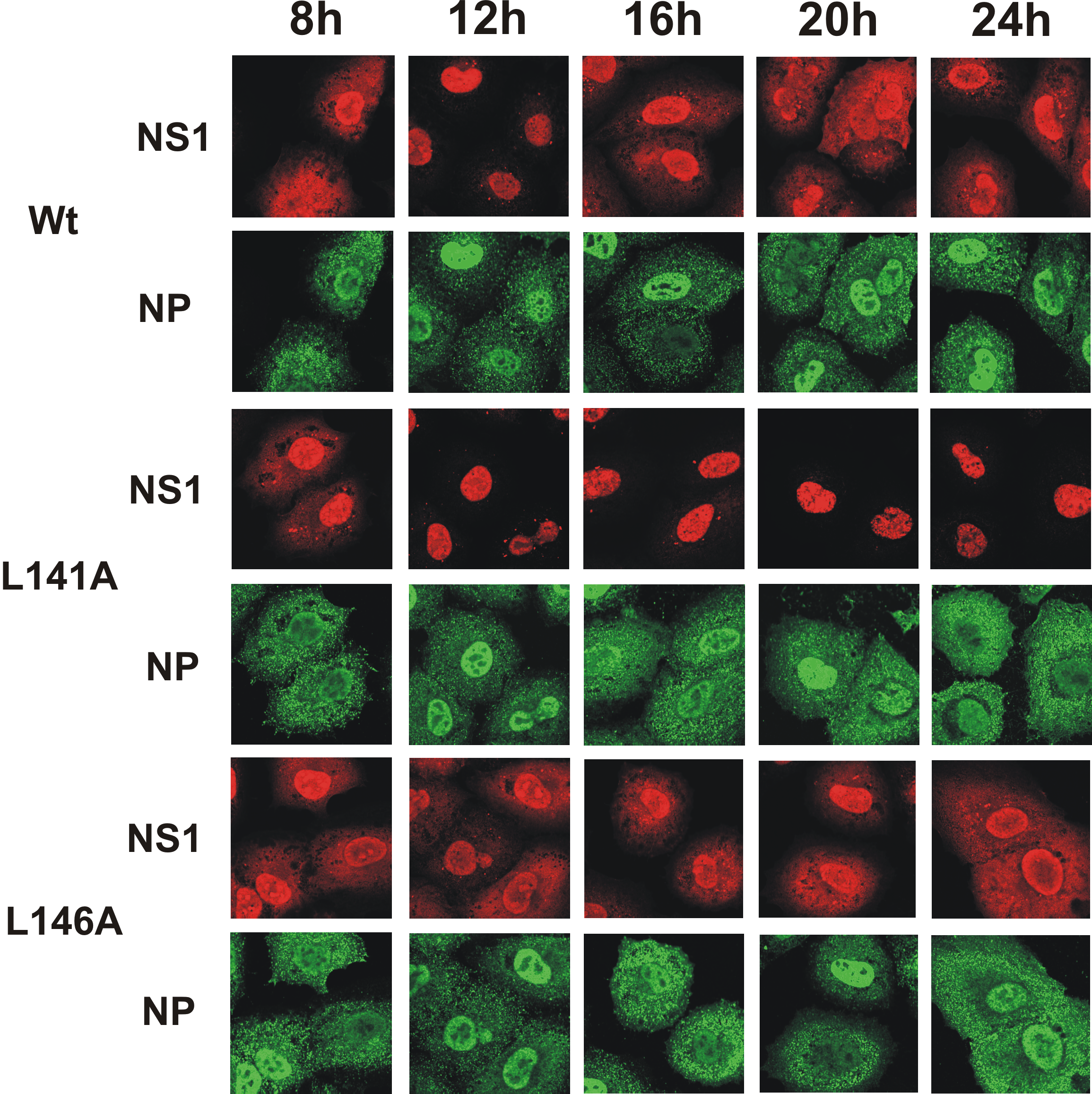

Supplement: Additional file 1: Figure S1 — Intracellular localization of NP and NS1 on A549 cells. A549 cells were infected at MOI 1 for the times indicated with wild type, (L141A) and (L146A) recombinant viruses before fixation and permeabilization. Cells were stained with guinea pig anti-NS1 and rabbit anti-NP antibodies followed by Rhodamine Red X-labeled anti-guinea pig immunoglobulins and FITC-labeled anti-rabbit immunoglobulins. Pictures were taken with Leica TCS NT confocal microscope. [file 1743-422X-11-128-S1.tiff]

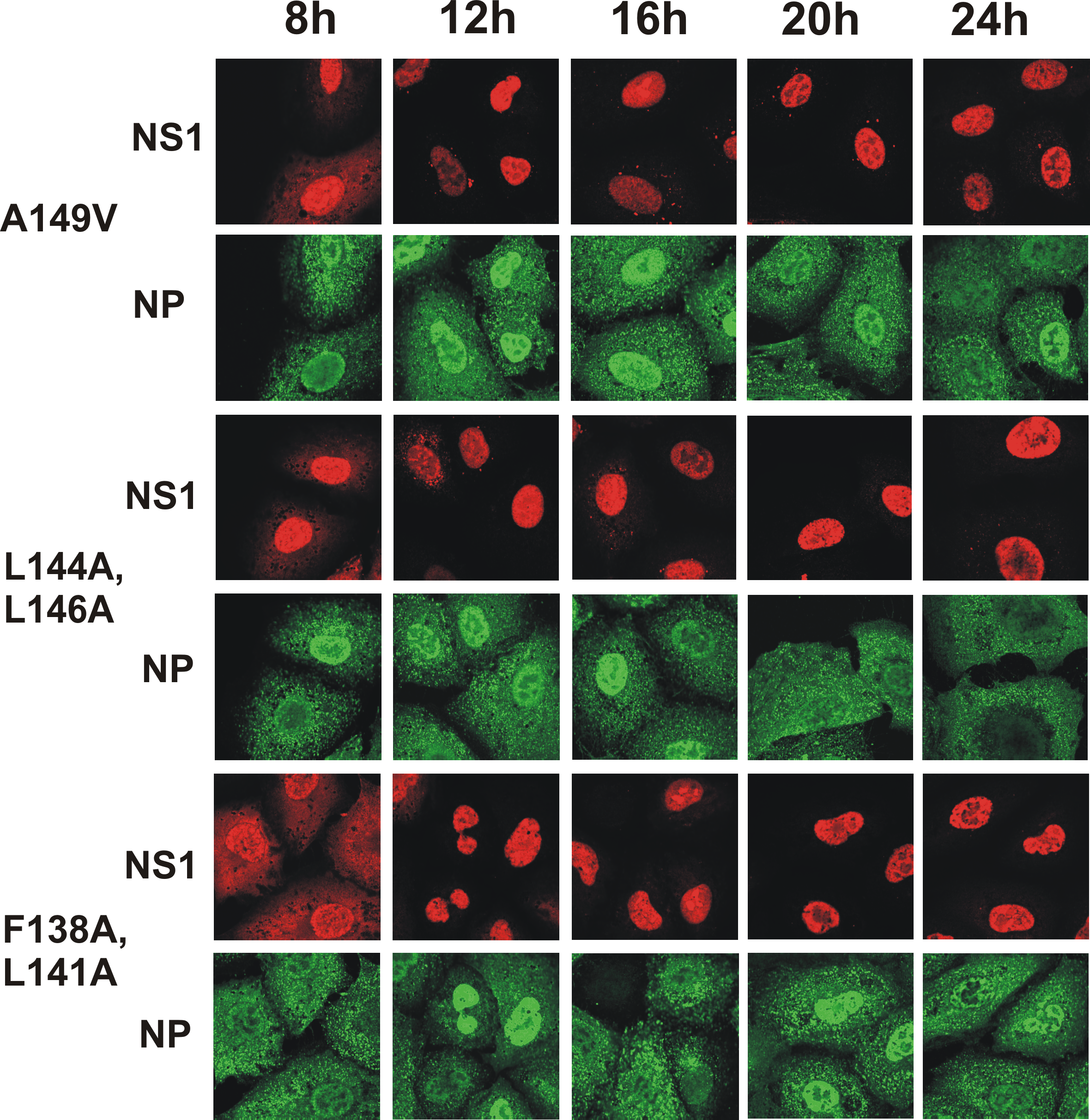

Supplement: Additional file 2: Figure S2 — Intracellular localization of NP and NS1 on A549 cells. A549 cells were infected at MOI 1 for the times indicated with (A149V), (L144A, L146A) and (F138A, L141A) recombinant viruses before fixation and permeabilization. Cells were stained with guinea pig anti-NS1 and rabbit anti-NP antibodies followed by Rhodamine Red X-labeled anti-guinea pig immunoglobulins and FITC-labeled anti-rabbit immunoglobulins. Pictures were taken with Leica TCS NT confocal microscope. [file 1743-422X-11-128-S2.tiff]

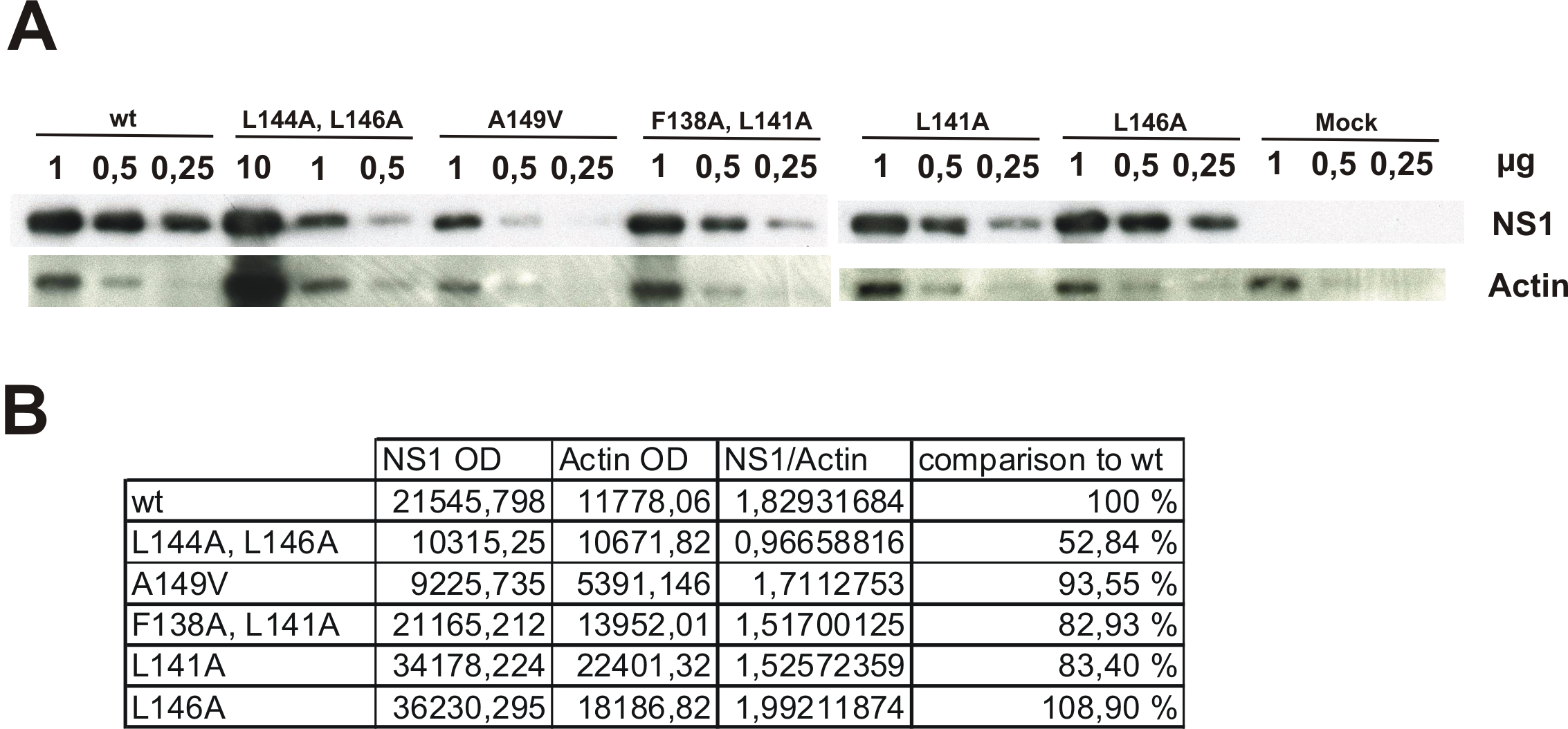

Supplement: Additional file 3: Figure S3 — Comparison of NS1 expression levels. A, lysates collected from infected A549 cells (MOI 1) at 20 h post-infection were analyzed by SDS-PAGE and Western blotting and the amounts of NS1 and actin were visualized by immunoblotting. Shown are three dilutions for each sample (10, 1, 0,5 or 0,25 μg total protein/lane). B, bands from the 1 μg lanes were processed with ImageJ software (http://imagej.nih.gov/ij/) to turn pixel intensity into optical density (OD). NS1 OD values were normalized to actin OD values and compared to wild type virus to get a relative estimate of NS1 expression levels between the viruses. Data shown is representative of two independent experiments. [file 1743-422X-11-128-S3.tiff]
